# Association between glycosylated haemoglobin and outcomes for patients discharged from hospital with diabetes: A health informatics approach

**DOI:** 10.1177/20552076211007661

**Published:** 2021-04-17

**Authors:** Tim Robbins, Sailesh Sankaranarayanan, Harpal Randeva, Sarah N Lim Choi Keung, Theodoros N Arvanitis

**Affiliations:** 1 University Hospitals Coventry & Warwickshire NHS Trust, Coventry, UK; 2 Institute of Digital Healthcare, WMG, University of Warwick, Coventry, UK; 3 Warwick Medical School, University of Warwick, Coventry, UK

**Keywords:** Diabetes, biochemistry, health informatics, hospital discharge, readmission

## Abstract

**Aims/Objectives:**

Extensive research considers associations between inpatient glycaemic control and outcomes during hospital admission; this cautions against overly tight glycaemic targets. Little research considers glycaemic control following hospital discharge. This is despite a clear understanding that people with diabetes are at increased risk of negative outcomes, following discharge. We evaluate absolute and relative Hba1c values, and frequency of Hba1c monitoring, on readmission and mortality rates for people discharged from hospital with diabetes.

**Methods:**

All discharges (n = 46,357) with diabetes from a major tertiary referral centre over 3 years were extracted, including biochemistry data. We conducted an evaluation of association between Hba1c, mortality and readmission, statistical significance and standardised Cohen’s D effect size calculations.

**Results:**

399 patients had a Hba1c performed during their admission. 3,138 patients had a Hba1c within 1 year of discharge. Mean average Hba1c for readmissions was 57.82 vs 60.39 for not readmitted (p = 0.009, Cohen’s D 0.28). Mean average number of days to Hba1c testing in readmitted was 97 vs 113 for those not readmitted (p = 0.00006, Cohen’s D 0.39). Further evaluation of mortality outcomes, cohorts of T1DM and T2DM and association of relative change in Hba1c was performed.

**Conclusions:**

Lower Hba1c values following discharge from hospital are significantly associated with increased risk of readmission, as is a shorter duration until testing. Similar patterns observed for mortality. Findings particularly prominent for T1DM. Further research needed to consider underlying causation and design of appropriate risk stratification models.

## Introduction

Diabetes represents a condition of impaired glycaemic control.^1^ Therefore, one of the central features of diabetes care aims to return glycaemic control into a physiological range through diet, oral medications or injectable medications.^2,3^ Glycaemic control is typically measured through either fingerpick blood sugar readings or glycosylated haemoglobin levels (Hba1c).^4^ The glycosylated haemoglobin value (Hba1c) reports the amount of glucose bound to haemoglobin molecules, and represents an average of blood sugar control over the preceding 6 weeks prior to the test being taken.^4^

There are not “normal values” for Hba1c as such, however the Hba1c value can be used in both the diagnosis of T2DM and monitoring of all types diabetes (although less useful in monitoring gestational diabetes mellitus – GDM. When monitoring control of diabetes, using Hba1c, it is typically the change in Hba1c value over time that is most useful rather than an individual Hba1c value. NICE generally recommend an Hba1c level target of 48 mmol/mol in treatment of T2DM. However, they stress that this target must be personalised and should be relaxed to 53 mmol/mol, where there is a risk of hypoglycaemia.

There is good evidence that maintaining glycaemic levels within physiological levels can reduce or minimise the risk of diabetic complications, in the long term (months-years), both for patients with type 1 diabetes and type 2 diabetes.^5,6^ Shorter term blood sugar control can also have a significant impact on health outcomes, with significantly higher or lower readings resulting in significant morbidity, mortality and healthcare utilisation.

Managing glycaemic control in the context of inter-current illness and diabetes is, a relatively complex process. In particular, there has been significant research considering the optimal glycaemic control for patients who are inpatients within hospital settings. There is a need to balance the risks of overly tight glycaemic control that risks hypoglycaemia versus the risks of thrombosis, diabetic ketoacidosis and hyperosmolar hyperglycaemic state that can occur with higher blood sugar values. It has been identified that glycaemic control can impact on both survival and length of stay for people with diabetes admitted to hospital.^7^ Hyperglycaemia has clearly been associated with adverse patient outcomes across a number of studies.^8,9^ However, interventions that have aimed to correct blood sugars into normal ranges have either not improved outcomes^10,11^ or in certain circumstances have led to worsening outcomes.^11^ Randomised controlled trials have suggested that hypoglycaemia is the primary driver of worsening patient outcomes associated with overly intense inpatient blood sugar control.^12^ Therefore, the overall consensus and guideline driven position for inpatient glycaemic control is that in general inpatient populations, a moderate, rather than overly tight control is advisable to optimise patient outcomes, including length of stay and readmission risk.^13^

However, when considering the impact of glycaemic control on the discharge process from hospital, and associated risks of readmission or mortality there has been much less research. Four articles consider the impact of glycaemic control in general on readmission; one of which focuses on the importance of the “most extreme blood sugar value” during inpatient admission and the second article considers the impact of glycaemic variability. These articles, therefore, considered relatively specific markers of inpatient glycaemic control and both were restricted to specific subsets of hospital inpatients with diabetes.^14–17^ The Diabetes Early Re-admission Risk Indicator (DERRI) is an externally validated and important tool that aims to predict all cause readmission within 30 days for patients discharged from hospital with diabetes, and found Hba1c to be significantly associated with readmission,^18^ the paper however notes that adding HbA1c to DERRI did not increase the accuracy of the model.

Importantly, however, there has been considerably more research looking at the impact of inpatient hypoglycaemia on readmission patterns for patients discharged from hospital with diabetes.^19–23^ These studies all considered generalised populations of people with diabetes admitted to hospital, rather than specific subsets of patients. It is likely that this focus on hypoglycaemia and readmission patterns is driven by an awareness of hypoglycaemia as a major driver of hospital admission and, therefore, cost in diabetes management. Remarkably, there is even less research considering the impact of glycaemic control on mortality outcomes following hospital discharge.

This paper looks to perform the first evaluation of the impact of glycaemic control on discharge outcomes of mortality and readmission, when patients with diabetes are discharged from hospital. It focuses on both the value of glycosylated haemoglobin and the frequency of monitoring. The use of Hba1c is selected due to its ready availability in electronic health record systems. As such it facilitates an informatics-based approach both in this research, but also when considering wider dissemination and adoption of this work in other settings.

## Methods

The study adopted a retrospective evaluation of data extracted from the electronic health record (EHR) of a large tertiary referral centre, in the West Midlands region of the United Kingdom, for all patients discharged from University Hospitals Coventry and Warwickshire NHS Trust with a diagnosis of diabetes, over a 3-year period. Only adult patients with Type 1 or Type 2 diabetes were included. Patients with GDM were excluded from the study. This is because Hba1c values vary significantly during pregnancy, with no clear normal ranges established.^24^ Indeed, the National Institute of Health & Clinical Excellence do not recommend Hba1c measurement during pregnancy.^25^

All Hba1c values for patients in the region (including those performed in the community setting) are analysed at the hospital laboratory and included within the electronic health record. Hba1c values were extracted for all patients discharged with diabetes as above. Patients from outside the region may have had Hba1c values calculated at other hospital laboratories and whose readings would not appear on University Hospital Coventry & Warwickshire NHS Trusts Electronic Health Record. Therefore, patients, who had postcode sectors outside of the Coventry & Warwickshire region, were also excluded from the study. Extraction of data was supported by a Biochemistry Performance and Programme Management Office Analyst.

The outcomes of interest were readmission within 30 days and mortality within 365 days. Multiple discharges were included per patient if they were admitted more than once. The association between Hba1c absolute values and frequency of Hba1c monitoring was analysed using Student’s T-Test, following adequate assessment for skew and kurtosis to ensure normality. An absolute skew value larger than 2 or an absolute kurtosis (proper) larger than 7 may be used as reference value for determining substantial non-normality. A p-value of <0.05 was considered significant. Standardised size was evaluated using Cohen’s D for pre-specified patient cohorts of patients with Type 1 Diabetes and Patients with Type 2 diabetes.

Ethical approval was granted by the local NHS Trust Research Ethics Committee, at University Hospitals Coventry & Warwickshire NHS Trust through the Governance arrangements for Research Ethics Committee Process [Study Ref: GF0220]. Approval was also granted through the University of Warwick’s Biomedical & Scientific Research Ethics Committee [Study Ref: REGO-2017-2114].

All statistical testing was performed using Microsoft Excel 2016^26^ and IBM’s SPSS v24.^27^

## Results

### Hba1c during admission

There were 399 patients meeting the inclusion criteria described above, who had a Hba1c sample analysed and recorded in the electronic health record system, during their hospital admission prior to discharge. 52 of these patients were readmitted within 30 days and 63 died within 365 days. The mean average Hba1c value of this cohort overall was 73 mmol/mol.

### Hba1c during admission – Readmission (Table 1)

The mean average Hba1c, during admission for patients, who were not readmitted within 30 days, was 74.6 mmol/mol, compared to 65.6 mmol/mol for patients readmitted within 30 days of discharge (p = 0.006, Cohen’s D 0.33).

**Table 1.** Association of Hba1c during admission with readmission (generalised population of patients with diabetes).

Average Hba1c during admission	n	Av Hba1c (mmol/mol)	Skew	Kurtosis
Not readmitted within 30 days	309	74.58	1.10	1.29
Readmitted within 30 days	52	65.62	1.78	3.98
P-value	0.0058			
Cohen's D	0.33			

### Hba1c during admission – Mortality (Table 2)

The mean average Hba1c, assessed during admission for patients who survived 365 days, was 69.05 mmol/mol and the mean average Hba1c, for patients who died within 365 days, was 64.11 mmol/mol. (p = 0.1, Cohen’s D N/A).

**Table 2.** Association of Hba1c during admission with mortality (generalised population of patients with diabetes).

Average Hba1c during admission	n	Av Hba1c (mmol/mol)	Skew	Kurtosis
No death within 365 days	305	69.05	0.99	0.75
Died within 365 days	56	64.11	2.19	6.37
P-value	0.10			
Cohen's D	0.21			

### Hba1c post discharge

For patients, who had an Hba1c assessed within a year of hospital discharge, the mean average Hba1c was 59.9 mmol/mol, with an average time until Hba1c assessment of 110 days.

### Hba1c post discharge readmission

When considering absolute Hba1c values and readmission for generalised populations of patients with diabetes (n = 3,403), the average Hba1c value of those not readmitted to hospital within 30 days was 60.4 mmol/mol, whereas the average Hba1c of those readmitted to hospital was 57.8 mmol/mol (p = 0.008, Cohen’s D 0.28) (Table 3).

**Table 3.** Association of Hba1c post-discharge with readmission (generalised population of patients with diabetes).

Average Hba1c post-discharge (All)	n	Av Hba1c (mmol/mol)	Skew	Kurtosis
Not readmitted within 30 days	2618	60.39	1.50	3.60
Readmitted within 30 days	520	57.82	1.75	4.15
P-value	0.0088			
Cohen's D	0.28			

The average number of days to Hba1c testing, for those discharged from hospital and not readmitted, was 115.23, whereas the average number of days until testing for those discharged and then readmitted was 83.05 days. (p < 0.001, Cohen’s D 0.39) (Table 4).

**Table 4.** Association between readmission and time to testing Hba1c (generalised population of people with diabetes).

Average time to hba1c post-discharge (All)	N	Average No. of days	Skew	Kurtosis
No. of days to test, no readmission within 30d	2618	115.23	0.80	–0.18
No. of days test , readmission within 30d	520	83.05	1.01	0.34
P-value	0.00006			
Cohen's D	0.39			

For patients with type 1 diabetes, the average Hba1c for those not readmitted was 74.4 mmol/mol, whereas the average Hba1c for those readmitted was 63.6 mmol/mol (p = 0.0077, Cohen’s D 0.44) (Table 5).

**Table 5.** Association of Hba1c post-discharge with readmission (T1DM population).

Average Hba1c post-discharge (T1DM)	n	Av Hba1c (mmol/mol)	Skew	Kurtosis
Not readmitted within 30 days	281	74.37	1.39	4.13
Readmitted within 30 days	42	63.64	0.86	0.35
P-value	0.0077			
Cohen's D	0.44			

The average number of days, between hospital discharge and the Hba1c being tested, was 109.4 for those readmitted and 114.9 for those not readmitted (p = 0.72, Cohen’s D N/A) (Table 6).

**Table 6.** Association between readmission and time to testing Hba1c (T1DM).

Average time to hba1c post-discharge (T1DM)	N	Average No. of days	Skew	Kurtosis
No. of days to test, no readmission within 30d	281	109.46	0.89	–0.21
No. of days to test, readmission within 30d	42	114.86	0.76	–0.54
P-value	0.72			
Cohen's D	N/A			

For patients with type 2 diabetes, the average Hba1c for those not readmitted was 58.8 mmol/mol, whereas the average Hba1c for those readmitted was 57.5 mmol/mol (p = 0.19, Cohen’s D N/A) (Table 7).

**Table 7.** Association of Hba1c post-discharge with readmission (T2DM population).

Average Hba1c post-discharge (T2DM)	n	Av Hba1c (mmol/mol)	Skew	Kurtosis
Not readmitted within 30 days	2246	58.83	1.45	2.59
Readmitted within 30 days	459	57.50	1.88	4.95
P-value	0.19			
Cohen's D	N/A			

The average number of days, between discharge and Hba1c being tested for those not readmitted, was 113.5 days, compared to 95.2 days for those readmitted (p < 0.001, Cohen’s D 0.33) (Table 8).

**Table 8.** Association between readmission and time to testing Hba1c (T2DM).

Average time to hba1c post-discharge (T2DM)	n	No. of days	Skew	Kurtosis
No. of days to test, no readmission within 30d	2246	113.50	0.80	–0.23
No. of days to test, readmission within 30d	459	95.23	1.05	0.50
P-value	0.00002			
Cohen's D	0.33			

### Hba1c post discharge mortality

The average Hba1c for the generalised population of patients with diabetes, who were discharged and survived for over one year, was 60.2 mmol/mol, whereas the average Hba1c for those with mortality within 1 year was 56.7 mmol/mol (p = 0.007, Cohen’s D 0.18) (Table 9).

**Table 9.** Association of Hba1c post-discharge with mortality (generalised population of patients with diabetes.

Average Hba1c post-discharge (All)	n	Av Hba1c (mmol/mol)	Skew	Kurtosis
No death within 365 days	2924	60.206	1.54	3.57
Died within 365 days	214	56.73	1.28	1.97
P-value	0.0074			
Cohen's D	0.18			

The mean average time to Hba1c testing, for those who survived over a year, was 112 days, whereas the mean average time to testing, for those with mortality within 1 year, was 83 days (p < 0.001, Cohen’s D 0.37) (Table 10).

**Table 10.** Association between mortality and time to testing Hba1c (generalised population of people with diabetes).

Average time to hba1c post-discharge	n	No. of days	Skew	Kurtosis
No. of days to test, survived 365 days	2924	112.19	0.81	–0.21
No. of days to test, died within 365 days	214	83.33	1.17	0.93
P-value	<0.0001			
Cohen's D	0.36			

For patients with type 1 diabetes, the average Hba1c for those who survived to 365 days, post discharge, was 73.2 mmol/mol, whereas the average Hba1c for those readmitted was 59.4 mmol/mol (p < 0.001, Cohen’s D 0.78) (Table 11).

**Table 11.** Association of Hba1c post-discharge with mortality (T1DM population).

Average Hba1c post-discharge (T1DM)	n	Av Hba1c (mmol/mol)	Skew	Kurtosis
No death within 365 days	308	73.20	1.27	3.58
Died within 365 days	15	59.41	–0.26	–1.57
P-value	0.00023			
Cohen's D	0.78			

The average number of days, between hospital discharge and the Hba1c being tested, was 110.8 for those readmitted and 98.5 for those not readmitted (p = 0.59, Cohen’s D N/A) (Table 12).

**Table 12.** Association between mortality and time to testing Hba1c (T1DM).

Average time to hba1c post-discharge (T1DM)	n	No. of days	Skew	Kurtosis
No. of days to test, survived 365 days	308	110.89	0.87	–0.26
No. of days to test, died within 365 days	15	98.47	0.73	–0.70
P-value	0.59			
Cohen's D	N/A			

For patients with type 2 diabetes, the average Hba1c, for those who survived 365 days post discharge, was 58.3, whereas the average Hba1c for those who died within 365 days was 54.2 (p = 0.07, Cohen’s D N/A) (Table 13).

**Table 13.** Association of Hba1c post-discharge with mortality (T2DM population).

Average Hba1c post-discharge (T2DM)	n	Av Hba1c (mmol/mol)	Skew	Kurtosis
No death within 365 days	2513	58.30	1.54	3.08
Died within 365 days	192	54.18	1.31	2.01
P-value	0.07			
Cohen's D	N/A			

The average number of days between discharge and Hba1c being tested, for those not readmitted, was 113.5 days, compared to 96.119 days for those readmitted (p < 0.001) Cohen’s D 0.21) (Table 14).

**Table 14.** Association between mortality and time to testing Hba1c (T2DM).

Average time to hba1c post-discharge (T2DM)	n	No. of days	Skew	Kurtosis
No. of days to test, survived 365 days	2513	113.53	0.80	–0.18
No. of days to test, died within 365 days	192	96.1	1.01	0.34
P-value	<0.0001			
Cohen's D	0.21			

## Discussion

The measurement of Hba1c, in patients with diabetes, has been a mainstay of monitoring disease and the long-term future risk of microvascular and microvascular risk since the Diabetes Control and Complications Trial (DCCT) and UK Prospective Diabetes Study: clinical and therapeutic implications for type 2 diabetes (UKPDS) published their results.^5,28^ In both these studies, increased Hba1c values are associated with higher levels of adverse outcomes within the patient populations. The research, reported in this paper, represents the first evaluation of the association, between glycaemic control on discharge outcomes of mortality and readmission, when patients with diabetes are discharged from hospital.

The results demonstrated no statistically significant associations between the Hba1c values, recorded during the inpatient stay, for both readmission and mortality outcomes. It is important to note that the population size was relatively small (361 patients) for each and may, thus, contribute towards the results not reaching significance. This small population size is notable, in that it potentially reflects a large proportion of patients with diabetes attending hospital but not having their Hba1c assessed during the admission period. This does not necessarily mean that clinical teams were not conscious of the Hba1c measure, when seeing these patients, but they may have considered the most recent Hba1c previously collected in the community setting. Additionally, the Hba1c values typically take, the laboratory at UHCW, 24 hours to process, therefore this delay may mean clinical teams feel there is less need to request an Hba1c value, for short admission patients.

However, statistically significant associations were noted in relation to Hba1c values following discharge from hospital. The Hba1c value is statistically significantly associated with 30-day readmission and 365-day mortality, in generalised populations of patients with diabetes. The Hba1c is statistically significantly associated with readmission and mortality for T1DM cohorts but this was not seen for T2DM cohorts. Importantly, for both the generalised population of patients with diabetes and the T1DM cohorts, it was a higher Hba1c that was associated with lower rates of mortality and readmission. This may seem counterintuitive; however, similar patterns were seen in inpatient studies, based on finger-prick based glucose readings. In these studies, higher blood sugar readings were protective.^29^ This was explained by the high risks of negative outcomes associated with hypoglycaemia, resulting from overly tight glycaemic control. It is likely that similar patterns may be being observed here, with hypoglycaemia already known to be a major driver of hospital readmission^30^ and mortality.^31^ It is possible that T2DM patients, who are on insulin or gliclazide, and T1DM (who are all on insulin) are the drivers of the results observed here, for both the generalised population of patients with diabetes and T1DM cohort. The high number of T2DM patients, who are not on hypoglycaemia inducing medications, may explain the lack of significance in this subpopulation. The medications patients are on is not extractable from the electronic patient record system used at UHCW NHS Trust and the source of data for this study. However, this is potentially and important observation and focus of future work, UHCW is in the process of procuring a new EHR system that includes electronic prescribing components and would facilitate future further work in this area.

The time, between discharge and the next testing of Hba1c values, was statistically significantly associated with both readmission and mortality, for generalised populations of patients with diabetes. The time, between discharge and testing, was not significantly associated with mortality or readmission for patients with T1DM; however, it was significantly associated with patients with T2DM. The pattern for the generalised diabetes cohort and T2DM cohort follows what would be anticipated, with negative outcomes associated with a shorter period to Hba1c measurement. This likely represents more frequent contact with medical team for patients with diabetes, who are more likely to experience negative outcomes, and these medical teams requesting Hba1c more frequently. The lack of statistically significant association for patients with T1DM may be explained either by the smaller sample size, or perhaps, more likely, by the more frequent contact these populations have with medical teams as a routine part of their care, regardless of their underlying risks. Indeed, nearly all T1DM are seen in secondary care hospital clinics, as opposed to T2DM cohorts who are managed in the community and, although meant to have an at least annual nurse review with Hba1c, they have significantly less contact with medical teams.

This study has a number of limitations. There is the potential for missing values and missing data. The number of Hba1c tests, performed during the inpatient admission period, is perhaps lower than expected. However, the extraction process was supported by a Biochemistry Analyst at a University Tertiary Centre and, therefore, likely reflects a full and complete dataset, as contained within the clinical system. Any informatics-based study risks issues with data availability and missing values and it would be important to repeat this study at other centres, in order to look for differences in the outcomes generated. The analysis of HbA1c values obtained within 1 year post discharge in relation to readmission at 30 days may include a substantial proportion of Hba1c values that were obtained after the readmission and are therefore not suited to developing risk prediction models, but do provide important information regarding the relationship between Hba1c and patient readmission risk. We did not consider Hba1c values recorded in the time period prior to admission to hospital, this could represent important future work, not only would it allow for increased sample sizes if this work was to be completed at a multicentre setting, but it would also enable early identification of patients at risk of readmission. For example, if the Hba1c value within the 3 months prior to admission was indeed a risk factor for readmission, active interventions could be planned throughout the admission period to reduce future risk.

Secondly, Hba1c values themselves can, to some extent be unreliable, primarily influenced by factors affecting the lifespan of a patient’s erythrocytes.^32^ Whilst, for the vast majority of patients being discharged from hospital, they are likely to represent a good marker of recent glycaemic control, in some circumstances, they can be misleading. Reasons for non-representative Hba1c values can include; blood transfusions,^33^ pregnancy,^24^ sickle cell disease,^34^ medications^35^ and dialysis.^36^ The most important of these perhaps being the impact of blood transfusions in patients discharged following surgery, trauma or gastrointestinal bleeding where large volumes of blood may have been transfused.

This informatics work is exploratory in nature. It is important to note that we have not adjusted for multiple observations (through processes such as generalized estimating equations), therefore there is a possibility that associations could be overestimated. Despite the exploratory nature of the work, it is nevertheless important in demonstrating that biochemistry data may have an important role in understanding risk for patients with diabetes. This is also highly relevant, as new technologies may allow earlier identification of patterns that have been suggested by the Hba1c results collected here. Hba1c retrospectively looks at glucose patterns over the previous 6-week period, acting as an average effect rather than simple “point-in-time” blood sugar readings, which are entirely dependent on when the blood sugar reading is actually performed (in hospital for example the majority of fingerpick testing may be done around acute decompensations or surgical interventions, during the inpatient procedure, thus giving a non-representative summary of the overall blood sugar profile). We are however now able to gain a better understanding of average blood sugar readings through continuous blood sugar monitoring systems (CGM)^37^ or interstitial fluid blood sugar monitoring systems, such as the Freestyle Libre.^38^ Data from these systems are not widely available, in relation to inpatient care and the immediate post discharge period. However, the research presented here suggests that such information may be of significant importance in better understanding the risks, when patients with diabetes are discharged from hospital.

Finally, this work stresses that the hospital discharge process is a continuum, not just a point in time, with Hba1c values stretching across that continuum. This is a particularly important observation and something that clinicians often forget.

## Conclusion

Glycaemic control is currently the main indicator of effective diabetes management; typically, this is assessed through Hba1c values. This paper creates new knowledge in demonstrating the association between glycaemic control in the post discharge period and negative outcomes of readmission and mortality both for generalised and specific subpopulations of diabetes. Importantly, this research extends current understanding from glycaemic control in the inpatient setting, where low Hba1c values may be associated with worse outcomes following hospital discharge, particularly for cohorts of patients with T1DM. This research represents an exploratory informatics-based work, identifying the need for further research to characterise the “peri-discharge” period from a glycaemic perspective. Newer technologies may enable a more detailed understanding of how glycaemic control varies, around the time of hospital discharge, and its subsequent influence on patient outcomes and thus could form the foundation of important future high-impact research.

## Availability of data and materials

Data was generated from the inpatient electronic health record patient-data of University Hospitals Coventry & Warwickshire NHS Foundation Trust. As is typical for data sets of this nature, whilst the information is anonymised, the ethical approval process requires analysis and storage of the raw data on secure NHS equipment due to the risk of inadvertent or indirect breeches to anonymization. The raw data may potentially be available from University Hospitals Coventry & Warwickshire NHS Trust subject to approval, ethical review and secure storage arrangements.

## References

[1] Kharroubi AT Darwish HM. Diabetes mellitus: the epidemic of the century. World J Diabetes 2015; 6: 850–867. 26131326 10.4239/wjd.v6.i6.850 PMC4478580

[2] Amiel SA Pursey N Higgins B, et al. Diagnosis and management of type 1 diabetes in adults: summary of updated NICE guidance. BMJ 2015; 351: h4188. 26311706 10.1136/bmj.h4188

[3] McGuire H Longson D Adler A, et al. Management of type 2 diabetes in adults: summary of updated NICE guidance. BMJ 2016; 353: i1575., 27052837 10.1136/bmj.i1575

[4] Sodi R McKay K Dampetla S, et al. Monitoring glycaemic control in patients with diabetes mellitus. BMJ 2018; 363: k4723. 30459149 10.1136/bmj.k4723

[5] Group DR. Diabetes control and complications trial (DCCT): update. Diabetes Care 1990; 13: 427–433. 2180661 10.2337/diacare.13.4.427

[6] King P Peacock I Donnelly R. The UK prospective diabetes study (UKPDS): clinical and therapeutic implications for type 2 diabetes. Br J Clin Pharmacol 1999; 48: 643–648. 10594464 10.1046/j.1365-2125.1999.00092.x PMC2014359

[7] Association AD. 14. Diabetes care in the hospital: standards of medical care in diabetes – 2018. Diabetes Care 2018; 41: S144–S151. 29222385 10.2337/dc18-S014

[8] Malmberg K Norhammar A Wedel H, et al. Glycometabolic state at admission: important risk marker of mortality in conventionally treated patients with diabetes mellitus and acute myocardial infarction: long-term results from the diabetes and Insulin-Glucose infusion in acute myocardial infarction (DIGAMI) study. Circulation 1999; 99: 2626–2632. 10338454 10.1161/01.cir.99.20.2626

[9] Clement S Braithwaite SS Magee MF, et al. Management of diabetes and hyperglycemia in hospitals. Diabetes Care 2004; 27: 553–591. 14747243 10.2337/diacare.27.2.553

[10] Wiener RS Wiener DC Larson RJ. Benefits and risks of tight glucose control in critically ill adults: a meta-analysis. JAMA 2008; 300: 933–944. 18728267 10.1001/jama.300.8.933

[11] Brunkhorst FM Engel C Bloos F, et al. Intensive insulin therapy and pentastarch resuscitation in severe sepsis. N Engl J Med 2008; 358: 125–139. 18184958 10.1056/NEJMoa070716

[12] Griesdale DE de Souza RJ van Dam RM, et al. Intensive insulin therapy and mortality among critically ill patients: a meta-analysis including NICE-SUGAR study data. CMAJ 2009; 180: 821–827. 19318387 10.1503/cmaj.090206 PMC2665940

[13] Association AD. 15. Diabetes care in the hospital: Standards of medical care in diabetes – 2019. Diabetes Care 2019; 42: S173–S181. 30559241 10.2337/dc19-S015

[14] Shohat N Foltz C Restrepo C, et al. Increased postoperative glucose variability is associated with adverse outcomes following orthopaedic surgery. Bone Joint J 2018; 100-B: 1125–1132. 30062937 10.1302/0301-620X.100B8.BJJ-2017-1283.R1

[15] Rubin DJ Golden SH McDonnell ME, et al. Predicting readmission risk of patients with diabetes hospitalized for cardiovascular disease: a retrospective cohort study. J Diabetes Complications 2017; 31: 1332–1339. 28571933 10.1016/j.jdiacomp.2017.04.021 PMC5512582

[16] Bates D Clark NG Cook RI, et al. American College of Endocrinology and American Association of Clinical Endocrinologists position statement on patient safety and medical system errors in diabetes and endocrinology. Endocrine Practice 2005; 11: 198–201. 10.4158/EP.11.3.197 16239208

[17] Karunakaran A Zhao H Rubin DJ. Predischarge and postdischarge risk factors for hospital readmission among patients with diabetes. Medical Care 2018; 56: 634–642. 29750681 10.1097/MLR.0000000000000931 PMC6082658

[18] Rubin DJ Recco D Turchin A, et al. External validation of the diabetes early re-admission risk indicator (Derri()). Endocr Pract 2018; 24: 527–541. 29624095 10.4158/EP-2018-0035 PMC6413863

[19] Lipska KJ Ross JS Wang Y, et al. National trends in US hospital admissions for hyperglycemia and hypoglycemia among medicare beneficiaries, 1999 to 2011. JAMA Intern Med 2014; 174: 1116–1124. 24838229 10.1001/jamainternmed.2014.1824 PMC4152370

[20] Kim H Ross JS Melkus GD, et al. Scheduled and unscheduled hospital readmissions among patients with diabetes. Am J Manag Care 2010; 16: 760–767. 20964472 PMC3024140

[21] Ena J Gómez-Huelgas R Gracia-Tello B, et al. Derivation and validation of a predictive model for the readmission of patients with diabetes mellitus treated in internal medicine departments. Revista Clínica Española (English Edition) 2018; 218: 271–278. 10.1016/j.rce.2018.03.010 29731294

[22] Raval AD Zhou S Wei W, et al. 30-Day readmission among elderly medicare beneficiaries with type 2 diabetes. Popul Health Manag 2015; 18: 256–264. 25608114 10.1089/pop.2014.0116 PMC4888086

[23] Spanakis EK Umpierrez GE Siddiqui T, et al. Association of glucose concentrations at hospital discharge with readmissions and mortality: a nationwide cohort study. The Journal of Clinical Endocrinology & Metabolism 2019; 104: 3679–3691. 31042288 10.1210/jc.2018-02575 PMC6642668

[24] Nielsen LR Ekbom P Damm P, et al. HbA1c levels are significantly lower in early and late pregnancy. Diabetes Care 2004; 27: 1200–1201. 15111545 10.2337/diacare.27.5.1200

[25] Walker J. NICE guidance on diabetes in pregnancy: management of diabetes and its complications from preconception to the postnatal period. NICE clinical guideline 63. London, March 2008. Diabet Med 2008; 25: 1025–1027. 19183306 10.1111/j.1464-5491.2008.02532.x

[26] Microsoft Corp. *MS Excel*. Redmond, WA, USA: Microsoft Corp., 2016.

[27] IBM. *SPSS Statistics for Windows.* Version 24.0. Armonk, NY: IBM: 2019.

[28] Stratton IM Adler AI Neil HAW, et al. Association of glycaemia with macrovascular and microvascular complications of type 2 diabetes (UKPDS 35): prospective observational study. BMJ 2000; 321: 405–412. 10938048 10.1136/bmj.321.7258.405 PMC27454

[29] Aron DC Tseng C-L Soroka O, et al. Balancing measures: identifying unintended consequences of diabetes quality performance measures in patients at high risk for hypoglycemia. Int J Qual Health Care 2019; 31: 246–251. 30053046 10.1093/intqhc/mzy151

[30] Wei N Wexler D Nathan D, et al. Intensification of diabetes medication and risk for 30‐day readmission. Diabet Med 2013; 30: e56–e62. 23126686 10.1111/dme.12061 PMC3552066

[31] Bonds DE Miller ME Bergenstal RM, et al. The association between symptomatic, severe hypoglycaemia and mortality in type 2 diabetes: retrospective epidemiological analysis of the ACCORD study. BMJ 2010; 340: b4909–b4909. 20061358 10.1136/bmj.b4909 PMC2803744

[32] Cohen RM Franco RS Khera PK, et al. Red cell life span heterogeneity in hematologically normal people is sufficient to alter HbA1c. Blood 2008; 112: 4284–4291. 18694998 10.1182/blood-2008-04-154112 PMC2581997

[33] Hellman R. When are HBA1C values misleading? AACE Clin Case Rep 2016; 2: e377–e379.

[34] Schnedl WJ Krause R Halwachs-Baumann G, et al. Evaluation of HbA1c determination methods in patients with hemoglobinopathies. Diabetes Care 2000; 23: 339–344. 10868862 10.2337/diacare.23.3.339

[35] Unnikrishnan R Anjana RM Mohan V. Drugs affecting HbA1c levels. Indian J Endocrinol Metab 2012; 16: 528–531. 22837911 10.4103/2230-8210.98004 PMC3401751

[36] O’Toole SM Fan SL Yaqoob MM, et al. Managing diabetes in dialysis patients. Postgrad Med J 2012; 88: 160–166. 22282737 10.1136/postgradmedj-2011-130354

[37] Bergenstal RM Beck RW Close KL, et al. Glucose management indicator (GMI): a new term for estimating A1C from continuous glucose monitoring. Diabetes Care 2018; 41: 2275–2280. 30224348 10.2337/dc18-1581 PMC6196826

[38] Fokkert M Van Dijk P Edens M, et al. Performance of the FreeStyle libre flash glucose monitoring system in patients with type 1 and 2 diabetes mellitus. BMJ Open Diabetes Res Care 2017; 5: e000320. 10.1136/bmjdrc-2016-000320 PMC5316912 28243449

